# Atlanta Society of Medicine

**Published:** 1899-08

**Authors:** 


### SOCIETY REPORTS.

---

### ATLANTA SOCIETY OF MEDICINE.

ATLANTA, GA., June 1, 1899.

*Regular Meeting of the Atlanta Society of Medicine.*

The meeting was called to order by the President, Dr. W. S. Goldsmith.

Dr. A. G. Hobbs, read a paper on "Some Remarks on Tact and Patience Necessary in Prognosis and Treatment of Chronic Deafness."

DISCUSSION.

*Dr. J. M. Crawford:* I enjoyed Dr. Hobbs's paper. It is a good one. The suggestions one would get by reading that paper carefully and slowly are very good indeed. The points as to treating the nose and throat or the nasopharynx in the treatment of ear troubles are very necessary. I don't think I ever saw a case of aural trouble *per se*, without it was from a foreign body in the outer portion of the ear. It always comes from an inflammation of the nose or throat. The pharyngeal mouth of the Eustachian tube at the junction of the nose or throat carries its inflammation directly to the ear.

As to the length of time of treatment of these cases of chronic aural troubles, I was taught for years that six weeks was long enough. The six weeks had to be carried out and no longer or shorter time would be given. In the last few years I have experimented along this line, and in one case especially I remember I saw the young man ninety-two times. That young man had previously been treated his six weeks on two or three different occasions, and probably by two or three different ones, and was told that was all that could be done for him. When he came to me the affection had been going on for a year and his hearing was my watch on pressure. This was not of a few years standing, or from a recent cold, but had been that way for several months. After treating him ninety-two times I examined his ear with the same watch under the same conditions, and he heard my watch at eight inches. It has been about seven months since I dismissed him as a daily patient, and I see him now but two or three times a month. I saw him Saturday, and his hearing is still eight inches. This shows how much we can accomplish by persistence; don't know whether you would call it tact or otherwise, but at least, patience and perseverance. What information I gained in that case shows that six weeks is not sufficient in treating an ear, as we were taught years ago.

Now, the doctor did not go into the method of treatment, and it might be presumptuous in me to say anything along that line, but if he will pardon me I will speak of the manner of inflation that I have been adopting for the last two years. It is with the compressed air apparatus instead of the Eustachian catheter and Politzer air bag. I tried three or four different methods, and I saw by the patient's knowledge of the difference which way the air entered the middle ear best; whether by the catheter or Politzer bag or compressed air apparatus. With the Politzer bag you cannot throw more than five pounds pressure through the Eustachian tube. With the compressed air apparatus you can use almost any pressure. I have used thirty-five pounds pressure and had no trouble with it. Instead of using the catheter and introducing through the nose, I have a nose-piece slipped on my compressed air tube and have the patient simply swallow or drink a little water, and just at the time of swallowing I press down upon the cut-off and then thirty-five pounds pressure goes through the nose into the middle ear. This is more effective than the bag and catheter. As said before, I have never had any danger from it and have never broken any drum membranes, even with children. Of course, we use it more cautiously with children. I have had better results by far than when I used the Politzer air bag.

*Dr. Hobbs* (closing the discussion): Do not know that I have anything further to say. I did not go into the method of treating these cases, as that would hardly come under this paper. However, I think Dr. Crawford uses a great deal of tact in applying thirty-five pounds pressure, and a great deal more tact in repeating it.

REPORT OF CASES.

*Dr. Hobbs:* I wish to report an interesting case of persistent recurrence of pieces of charcoal in the conjunctival sac. All these pieces of charcoal came from the eye of a little colored girl. The first piece got in the eye accidentally, or was placed there. No doubt all of you have had just such cases where they wanted to pose as martyrs, or perhaps they had some other reason for doing it. The first one I removed from the eye as a foreign body, supposing it had gotten there by accident. The next day she came back with two or three times as many, and I wondered how I could have overlooked them. I noticed that the eye was not much inflamed. She came back the third day and the fourth day with still more and larger ones, and then the eye began to get inflamed, and not till then. Ordinarily, you know, the eye is inflamed by the least foreign particle. She had been coming back day after day with these particles in her eye, and I had gotten tired of it, and I ordered her to stop it. I told her mother that when she brought her back next day there would be no more in the eye unless she put them there herself. When she was brought back the next day there were not any more in the eye, although it was still inflamed. The peculiar part of the case was that she would put them in her eye and yet her mother did not know anything about it or suspect it. Also, it was a very unusual case of resistance of the conjunctiva, as it did not become inflamed until the fourth day. As to the reason for the child doing as she did, perhaps it may have been due to her environments, but this family is an intelligent colored family, and well-to-do. However, she had some trouble with her eyes before, and had been treated for it, and she frankly told me afterward that she really liked the sensation of being treated, and this may have been the reason for putting these in her eye each day. The girl was fifteen years of age.

*Dr. Hutchins:* Dr. Hobbs's case reminds me of one that I had lately, but the trouble was not in the eye. It was another kind of playing-off, so I heard. Last fall a girl sixteen years of age was brought to me with a history of a broken finger, and upon examination I found the skin was cracked over the last joint of the little finger. She complained of intense pain on pressure, and yet there was no evidence of disease except the crack in the finger. She got some better, but afterwards went the round of the doctors and came back to me with the finger cracked as bad as ever, and complained of pain in the entire finger and pain all the way up the arm. The mother told me that it had broken out in black and blue spots, which had disappeared immediately. This was a new disease to me. One day a lady came with her and told me that the girl was naturally stupid, and that she was playing off to escape having to go to school. This was about the best explanation of the trouble, and I am inclined to think that it was a case of self-inflicted injury.

---

### DR. BRANSFORD'S HEROISM RECOGNIZED.

Passed Assistant Surgeon J. W. Bransford, United States Navy, was advanced three numbers by the last Congress for heroism in the naval battle of Santiago. Surgeon Bransford was attached to the converted yacht Gloucester, which destroyed the Spanish torpedo-boat-destroyers, and, having no wounded to demand his care, he took charge of one of the rapid-fire guns with marked effect, his conduct eliciting the official praise of his commanding officer. Dr. Bransford had served eighteen years as an assisnant surgeon in the navy, but had afterward resigned and was in civil practice when the war broke out. He promptly offered his services and was given a volunteer commission. A bill to appoint him an assistant surgeon in the navy and place him on the retired list passed the Senate, but failed to receive action by the House during the more pressing matters which occupied the latter part of its session.—*Boston Medical and Surgical Journal.*

---

### CONGENTAL ABSENCE OF BOTH EYES.

Guerrant (*Archives of Pediatrics*) reports the case of primipara at full term who gave birth to a male child with congenital absence of both eyes. There was no trace of globe in either orbit. The orbital fissures were about one-fourth inch in length. There was no orbital enormity, and the forehead was not high. In other ways the child was normally developed.

